# Nrf2 is activated by disruption of mitochondrial thiol homeostasis but not by enhanced mitochondrial superoxide production

**DOI:** 10.1074/jbc.RA120.016551

**Published:** 2020-12-13

**Authors:** Filip Cvetko, Stuart T. Caldwell, Maureen Higgins, Takafumi Suzuki, Masayuki Yamamoto, Hiran A. Prag, Richard C. Hartley, Albena T. Dinkova-Kostova, Michael P. Murphy

**Affiliations:** 1 MRC Mitochondrial Biology Unit, University of Cambridge, Cambridge, UK; 2 School of Chemistry, University of Glasgow, Glasgow, UK; 3 Division of Cellular Medicine, School of Medicine, Jacqui Wood Cancer Centre, University of Dundee, Dundee, Scotland, UK; 4 Department of Medical Biochemistry, Tohoku University Graduate School of Medicine, Sendai, Japan; 5 Tohoku Medical Megabank Organization, Tohoku University, Sendai, Japan; 6 Department of Pharmacology and Molecular Sciences and Department of Medicine, Johns Hopkins University School of Medicine, Baltimore, Maryland, USA; 7 Department of Medicine, University of Cambridge, Cambridge, UK

**Keywords:** reactive oxygen species (ROS), MitoPQ, MitoCDNB, Nrf2, redox signaling, thiol oxidation, energy metabolism, ACN, acetonitrile, BSA, bovine serum albumin, CD, concentration required to double, CDNB, 1-chloro-2,4-dinitrobenzene, DRP1, dynamin-related protein 1, FB, fractionation buffer, GCLC, glutamate–cysteine ligase catalytic subunit, GSS, GSH synthetase, HO-1, heme oxygenase-1, Keap1, Kelch-like ECH-associated protein 1, MEF, mouse embryonic fibroblast, MitoCDNB, MitoChlorodinitrobenzoic acid, MitoPQ, MitoParaquat, NAC, *N*-acetyl-L-cysteine, NQO1, NAD(P)H:quinone oxidoreductase-1, Nrf2, nuclear factor erythroid 2–related factor 2, PGAM5, phosphoglycerate mutase family member 5, ROS, reactive oxygen species, SFN, sulforaphane, TRX, thioredoxin, TRXR2, TRX reductase 2

## Abstract

The transcription factor nuclear factor erythroid 2–related factor 2 (Nrf2) regulates the expression of genes involved in antioxidant defenses to modulate fundamental cellular processes such as mitochondrial function and GSH metabolism. Previous reports proposed that mitochondrial reactive oxygen species production and disruption of the GSH pool activate the Nrf2 pathway, suggesting that Nrf2 senses mitochondrial redox signals and/or oxidative damage and signals to the nucleus to respond appropriately. However, until now, it has not been possible to disentangle the overlapping effects of mitochondrial superoxide/hydrogen peroxide production as a redox signal from changes to mitochondrial thiol homeostasis on Nrf2. Recently, we developed mitochondria-targeted reagents that can independently induce mitochondrial superoxide and hydrogen peroxide production mitoParaquat (MitoPQ) or selectively disrupt mitochondrial thiol homeostasis MitoChlorodinitrobenzoic acid (MitoCDNB). Using these reagents, here we have determined how enhanced generation of mitochondrial superoxide and hydrogen peroxide or disruption of mitochondrial thiol homeostasis affects activation of the Nrf2 system in cells, which was assessed by the Nrf2 protein level, nuclear translocation, and expression of its target genes. We found that selective disruption of the mitochondrial GSH pool and inhibition of its thioredoxin system by MitoCDNB led to Nrf2 activation, whereas using MitoPQ to enhance the production of mitochondrial superoxide and hydrogen peroxide alone did not. We further showed that Nrf2 activation by MitoCDNB requires cysteine sensors of Kelch-like ECH-associated protein 1 (Keap1). These findings provide important information on how disruption to mitochondrial redox homeostasis is sensed in the cytoplasm and signaled to the nucleus.

Oxidative stress and damage are involved in the development and progression of many diseases (1, 2). Cells are equipped with elaborate defense systems that allow them to maintain homeostasis in the face of physiological stress. The transcription factor nuclear factor erythroid 2–related factor 2 (Nrf2) plays a central role in the cytoprotective response to oxidative stress and damage (3, 4). In unstressed conditions, Nrf2 protein levels are maintained relatively low, which is due to its constitutive ubiquitination mediated by Kelch-like ECH-associated protein 1 (Keap1), an adaptor component of a Cullin 3–based ubiquitin E3 ligase complex, which targets Nrf2 for proteasomal degradation (5, 6). Upon exposure to oxidants and/or electrophiles such as sulforaphane (SFN), specific cysteine sensors in Keap1 are modified, although the details of the specific reactions are not clear (7, 8, 9). This inhibits the ubiquitination of Nrf2, which in turn leads to the stabilization and accumulation of Nrf2 (10). Nrf2 then translocates to the nucleus where it acts as a transcription factor, binding to the antioxidant response elements in the promotor regions of Nrf2 target genes, upregulating the expression of a series of antioxidant genes (11, 12).

In addition to its role in overall cellular redox homeostasis, Nrf2 is also critical for the maintenance of mitochondrial antioxidant defenses and redox homeostasis (13). This is of particular importance because mitochondria are a major source of hydrogen peroxide due to superoxide production from respiratory complexes (14, 15). Within the mitochondrial matrix, this superoxide is rapidly converted by Mn superoxide dismutase to hydrogen peroxide, which can both contribute to oxidative damage in a range of pathologies, but also acts as a signaling molecule that transduces redox signals through modifying the activity of redox-sensitive proteins (16, 17, 18). There are many mitochondrial thiol redox systems that are independent from those in the cytosol (19, 20, 21) and include the organelle’s GSH and thioredoxin (TRX) systems, both of which are critical for cell viability and function (22, 23, 24). Mitochondrial GSH is made in the cytosol and then transported from the cytosol into the mitochondrial matrix where it is maintained in a reduced state by GSH reductase (24). GSH is used by GSH peroxidases 1 and 4, glutathione-S-transferases (GSTs) and glutaredoxin-2 to protect against reactive oxygen species (ROS), electrophiles, xenobiotics, and protein thiol oxidation (24). The mitochondrial TRX system consists of TRX2 and TRX reductase 2 (TRXR2), which maintains TRX2 in a reduced state by using mitochondrial NADPH as a substrate (25, 26). TRX2 maintains the activities of the peroxidase Peroxiredoxin 3 and of methionine sulfoxide reductases, while also directly reducing protein disulfides (27).

The Nrf2 activity enhances the expression of antioxidant systems (28, 29), including GSH synthesis (30), GSH peroxidases (31), GSH reductase (32), Peroxiredoxin 3 (33, 34, 35), and TRXR2 (33, 34). The Nrf2 activity also affects mitochondrial biogenesis by influencing the expression of critical transcription factors, such as peroxisome proliferator–activated receptor gamma (36). These responses enable mitochondria to adapt to elevated oxidative stress and damage; consequently, Nrf2 deficiency leads to mitochondrial damage (37). Therefore, it is widely assumed that Nrf2 is upregulated in response to mitochondrial oxidative stress and damage (28). However, the mechanistic details by which mitochondrial oxidative stress and damage activate Nrf2 are still unclear. Possibilities include that elevated mitochondrial superoxide production generates hydrogen peroxide that goes from the mitochondria to the cytosol to activate Nrf2 directly or indirectly. Alternatively, the redox changes within the mitochondria may lead to secondary signals to the cytosol that then activate Nrf2. Finally, there are also suggestions that mitochondrial dysfunction may activate Nrf2 through formation of a complex with Keap1 and the mitochondrial outer membrane serine/threonine protein phosphatase, phosphoglycerate mutase family member 5 (PGAM5) (28, 38, 39). This Nrf2–Keap1–PGAM5 complex has been proposed as an effector for ROS-induced necrosis and as an activator of mitochondrial fragmentation mediated through dephosphorylation of dynamin-related protein 1 (DRP1) (40). However, whether the role of Nrf2 bound to the mitochondrial outer membrane is distinct from that of the main cytosolic pool of Nrf2 is unclear.

In exploring how mitochondrial oxidative stress and damage activate Nrf2 (28), it has not been possible to distinguish between the effects of superoxide and hydrogen peroxide generation, and redox changes in mitochondria independently from those in the rest of the cell. Furthermore, many Nrf2 activators cause changes in both superoxide and hydrogen peroxide levels and in thiol homeostasis. However, these pathways interact closely and changes in thiol homeostasis can affect hydrogen peroxide levels, while conversely, increased levels of hydrogen peroxide can alter the thiol redox state *via* peroxidases (4, 12, 13). However, Nrf2 is regulated in different ways by these effectors, in part through the differential reactivity of particular thiols on Keap1 (41), suggesting that ROS such as hydrogen peroxide and thiol redox alterations affect Nrf2 differently. Therefore, our challenge was to address the role in Nrf2 activation of mitochondrial redox changes independently of those from the cytosol while also distinguishing between the impact of mitochondrial superoxide and hydrogen peroxide production, and that of thiol redox changes. To do this, we used two mitochondria-targeted redox active agents. To investigate thiol redox state, we used a mitochondria-targeted disrupter of thiol redox homeostasis MitoChlorodinitrobenzoic acid (MitoCDNB), a 1-chloro-2,4-dinitrobenzene (CDNB) derivative that is selectively taken up by mitochondria within cells where it selectively depletes mitochondrial GSH largely, but not solely, by acting as a substrate for mitochondrial GSTs while also inhibiting the mitochondrial Trx system by inhibiting TRX reductases (Fig. 1*A*) (42). In addition, we used mitoParaquat (MitoPQ), a mitochondria-targeted redox cycler, which is selectively taken up by mitochondria where the paraquat moiety reacts with the complex I flavin to selectively increase superoxide production by redox cycling and thus increase hydrogen peroxide within the mitochondrial matrix (Fig. 1*B*) (43, 44, 45). Our hypothesis was that inducing mitochondrial dysfunction through these two independent chemical biology approaches would provide insights into the redox signaling mechanisms that underlie activation of Nrf2 by mitochondrial oxidative stress and damage (Fig. 1). We found that disruption of the mitochondrial thiol homeostasis with MitoCDNB activated Nrf2, whereas enhancing mitochondrial superoxide production with MitoPQ did not. In addition, Nrf2 activation by MitoCDNB was greatly diminished by the thiol *N*-acetyl-*L*-cysteine (NAC) and in cells expressing mutant Keap1 that lacked particular sensor cysteine residues. These results indicate that elevated mitochondrial superoxide generation alone does not activate Nrf2 but provide confirmation of direct signaling to the cytosol as a stress response to disrupted mitochondrial thiol redox homeostasis.

**Figure 1:**
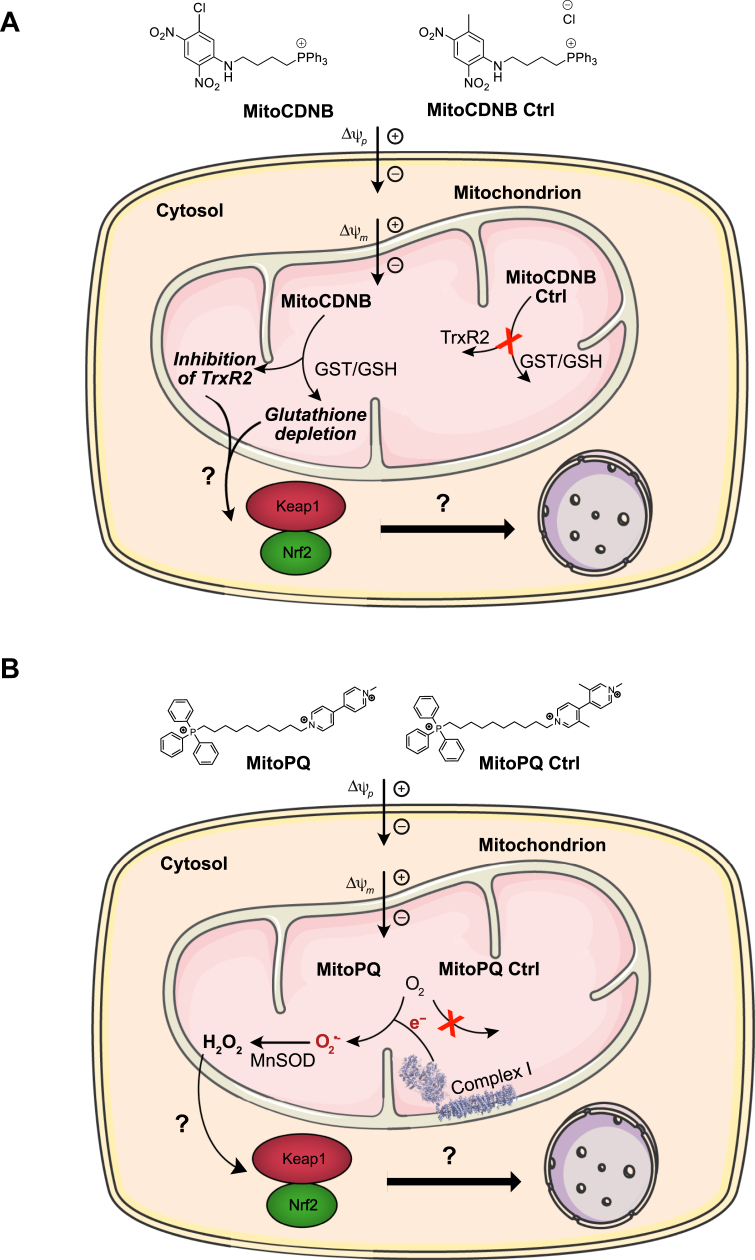
**Mode of action of MitoCDNB and MitoPQ.***A*, MitoCDNB is composed of a 1,5-dichloro-2,4-dinitrobenzene (CDNB) moiety and mitochondria-targeting triphenylphosphonium cation. The latter leads to its selective accumulation within the mitochondrial matrix, driven by the plasma and mitochondrial membrane potentials. Within the mitochondria, the CDNB moiety acts as a GST substrate to deplete GSH, and it is also a TrxR2 inhibitor, leading to the disruption of mitochondrial thiol redox defense homeostasis. We hypothesize this could lead to Nrf2 activation and its nuclear localization. Its inactive control compound, MitoChlorodinitrobenzoic acid (MitoCDNB) Ctrl accumulates in the mitochondrial matrix but does not lead to GSH depletion or TrxR2 inhibition. *B*, MitoParaquat (MitoPQ) is composed of a redox cycling paraquat moiety and a hydrophobic carbon chain linking it to a mitochondria-targeting triphenylphosphonium cation. MitoPQ is accumulated by mitochondria driven by the plasma and mitochondrial membrane potentials. Within the matrix, MitoPQ is reduced to a radical monocation by one-electron reduction at the flavin site of complex I, which subsequently interacts rapidly with O_2_ to generate superoxide (O_2_^•−^). We hypothesize the ability of mitochondrial-specific O_2_^•−^ production to activate the Keap1/Nrf2 pathway and lead to Nrf2 nuclear localization. The inactive control compound, MitoPQ Ctrl, selectively accumulates in the matrix but does not act as a redox cycler and therefore is unable to produce O_2_^•−^. Nrf2, nuclear factor erythroid 2–related factor 2; TRXR2, TRX reductase 2.

## Results and discussion

### Selective disruption of mitochondrial thiol homeostasis activates Nrf2

**Figure 2:**
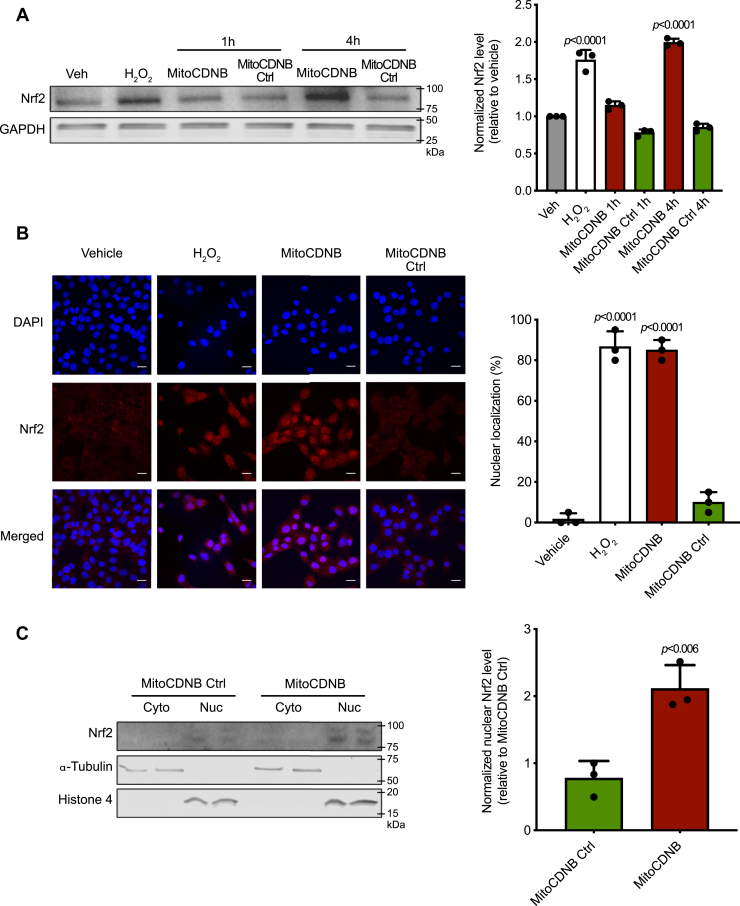
**Nrf2 protein levels and subcellular localization upon exposure to MitoCDNB.***A*, Western blotting of Nrf2 protein levels. C2C12 cells were incubated with a vehicle (0.1% ethanol; Veh), H_2_O_2_ (100 μM for 30 min), MitoChlorodinitrobenzoic acid (MitoCDNB) (10 μM for 1 and 4 h), or MitoCDNB Ctrl (10 μM for 1 and 4 h). Protein levels were assessed by Western blotting for Nrf2 (*top*) and GAPDH (*bottom*) in whole-cell lysates and band intensities quantified (*right*). *B*, 3D maximum projection images showing fluorescence obtained with C2C12 cells with DAPI nuclear staining (*first row*), immunocytochemistry for Nrf2 (*second row*), and composite merge of the two fluorescent channels (*third row*) after treatment of 100-μM H_2_O_2_ for 30 min or 10-μM MitoCDNB for 4 h and MitoCDNB Ctrl. Scale bars: 20 μm. Nuclear distribution (*right*) is presented as the mean percentage of all cells ±SD. Data are from 3 independent experiments; 30 cells were counted for each condition. *C*, C2C12 cells were incubated for 4 h with 10 μM of either MitoCDNB Ctrl or MitoCDNB and fractionated into cytosolic and nuclear fractions. Protein levels were assessed by Western blotting for Nrf2 (*top*), alpha-tubulin (*middle*), and histone-4 (*bottom*). All data are the mean ± SD. Blots are representative of three independent experiments. *p* values were calculated using one-way ANOVA (Tukey’s post hoc correction for multiple comparison) or two-tailed, unpaired Student’s t-test. Individual significant *p* values are shown. Nrf2, nuclear factor erythroid 2–related factor 2.

We first assessed if selective disruption of mitochondrial thiol redox homeostasis activated Nrf2. To do this, we used the mitochondria-targeted thiol reagent MitoCDNB, which we had previously shown selectively depletes GSH and inhibits TrxR2 within mitochondria, without directly affecting their cytosolic counterparts (42). Under control conditions, the Nrf2 protein is present at low levels and is only detectable in the cytosol of C2C12 mouse myoblasts (Fig. 2*A*). Treatment with H_2_O_2_ as a positive control (100 μM, 30 min) increased the protein levels of Nrf2 within the cell ∼2-fold (Fig. 2*A*) and caused its redistribution to the nucleus, as assessed by immunocytochemistry (Fig. 2*B*), as did the positive control SFN (Fig. S1). Similarly, MitoCDNB (10 μM, 4 h) increased Nrf2 protein levels ∼2-fold (Fig. 2*A*) and led to its translocation to the nucleus, as assessed by immunocytochemistry (Fig. 2*B*) and by subcellular fractionation (Fig. 2*C*), with nearly 90% of cells having a clear nuclear distribution of Nrf2 upon MitoCDNB treatment (Fig. 2*B*). To determine whether this effect of MitoCDNB was due to its reaction with mitochondrial thiols, or by a nonspecific disruption of mitochondria by accumulation of the triphenylphosphonium-targeting group, we synthesized a MitoCDNB control compound (Fig. S2). The control compound is structurally very similar to MitoCDNB and is accumulated by mitochondria in response to the membrane potential, but lacks thiol reactivity (Fig. S2, *A*–*C*). This control compound had no effect on Nrf2 protein levels or nuclear translocation (Fig. 2, *A*–*D*). Thus, the activation of Nrf2 by MitoCDNB is dependent on its thiol reactivity and not a nonspecific interaction of the mitochondria-targeting moiety.

**Figure 3:**
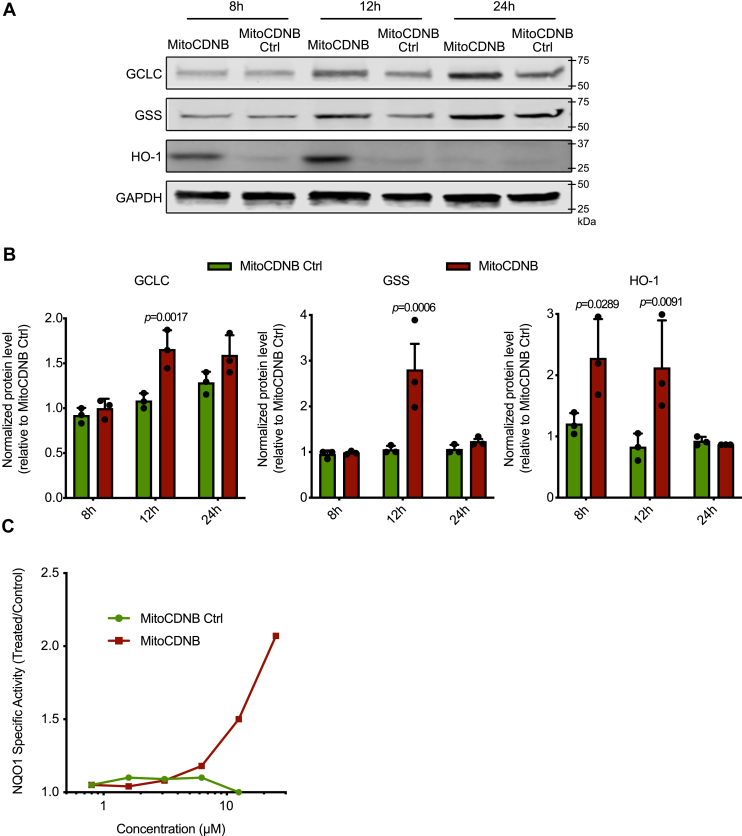
**MitoCDNB induces Nrf2 downstream targets.***A*, induction of Nrf2 downstream targets (GCLC, GSS, HO-1) after treatment with 10-μM MitoChlorodinitrobenzoic acid (MitoCDNB) and MitoCDNB Ctrl for 8, 12, or 24 h. Protein levels were assessed by Western blotting for GCLC, GSS, HO-1, and GAPDH. *B*, relative fold of induction was obtained as compared with the untreated/MitoCDNB Ctrl and GAPDH Western blots. Data are from 3 independent experiments. All data are the mean ± SD. Blots are representative of three independent experiments. *p* values were calculated using one-way ANOVA (Tukey’s post hoc correction for multiple comparisons) or two-tailed, unpaired Student’s T-test. Individual significant *p* values are shown. *C*, NQO1 activity in mouse Hepa1c1c7 cells treated with MitoCDNB or MitoCDNB Ctrl for 48 h (n = 8). The mean values are shown. GCLC, glutamate–cysteine ligase catalytic subunit; GSS, GSH synthetase; HO-1, heme oxygenase-1; NQO1, NAD(P)H:quinone oxidoreductase-1; Nrf2, nuclear factor erythroid 2–related factor 2.

To determine whether the nuclear accumulation of Nrf2 by MitoCDNB activates transcription of Nrf2-dependent genes, we assessed the levels of proteins known to be under Nrf2 control *via* the antioxidant response element. Immunoblotting showed that MitoCDNB, but not its corresponding control compound, led to a time-dependent increase in the levels of the Nrf2 downstream targets glutamate–cysteine ligase catalytic subunit (GCLC), GSH synthetase (GSS), and heme oxygenase-1 (HO-1). HO-1 was increased at 8 and 12 h after exposure, while GCLC and GSS levels increased later, at 12 h (Fig. 3, *A*–*B*). To further confirm Nrf2 activation, we used the quantitative NAD(P)H:quinone oxidoreductase-1 (NQO1) inducer assay (46, 47). The activity of this NAD(P)H:quinone oxidoreductase enzyme, which is involved in detoxification pathways, is a particularly sensitive indication of Nrf2 activation as transcription of its gene is primarily regulated by Nrf2, and thus, NQO1 is widely recognized as a classical Nrf2 target (48). The potency of MitoCDNB was defined as the concentration required to double (CD) the NQO1 enzyme-specific activity. For this Hepa1c1c7, cells were incubated with MitoCDNB, or its control, for 24 h, and NQO1 activity was assessed. MitoCDNB elicited a pronounced concentration-dependent NQO1 induction with a CD value of 12.5 μM that facilitates comparison of its potency with other inducers (Fig. 3*C*), whereas the control compound had no effect. We conclude that the selective disruption of mitochondrial thiol homeostasis by MitoCDNB activates Nrf2.

### Selective generation of superoxide within mitochondria does not activate Nrf2

**Figure 4:**
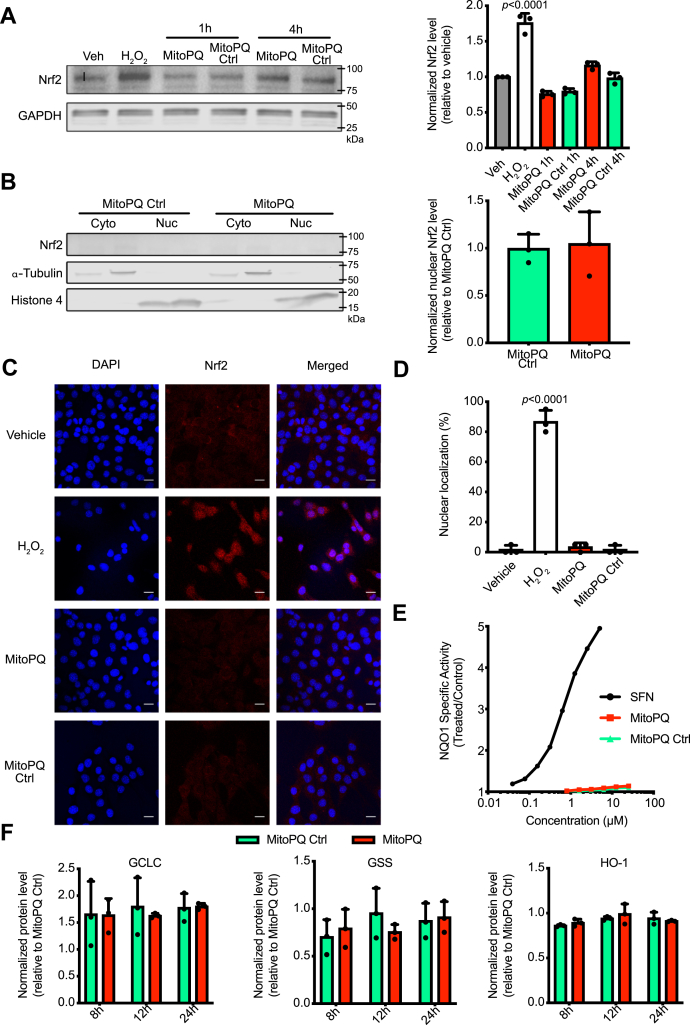
**MitoPQ effect on Nrf2 protein levels, its subcellular localization, and downstream targets.***A*, Western blotting of Nrf2 protein levels. C2C12 cells were incubated with a vehicle (0.1% ethanol; Veh), H_2_O_2_ (100 μM for 30 min), MitoParaquat (MitoPQ) (5 μM for 1 and 4 h), and MitoPQ Ctrl (5 μM for 1 and 4 h). Lysates were then assessed by immunoblotting for Nrf2 (*top*) and GAPDH (*bottom*). *B*, C2C12 cells were incubated for 4 h with 5 μM of either MitoPQ Ctrl or MitoPQ and fractionated into cytosolic and nuclear fractions. Protein levels were assessed by Western blotting for Nrf2 (*top*), alpha-tubulin (*middle*), and histone-4 (*bottom*). *C*, 3D maximum projection images showing fluorescence obtained with C2C12 cells with DAPI nuclear staining (*first column*), immunocytochemistry for Nrf2 (*second column*), and composite merge of the two fluorescent channels (*third column*). C2C12 cells were incubated with a vehicle (0.1% ethanol), MitoPQ Ctrl (5 μM), and MitoPQ (5 μM) for 4 h; 100-μM H_2_O_2_ for 30 min (same positive control as in Fig. 2*B*). Scale bars: 20 μm. *D*, quantification of nuclear distribution of Nrf2 in C2C12 cells after incubation with a vehicle, 100-μM H_2_O_2_, 5-μM MitoPQ and MitoPQ Ctrl. Nuclear distribution is presented as the mean percentage of all cells ±SD. Data are from 3 independent experiments; 30 cells were counted for each condition. *E*, NQO1 activity in mouse Hepa1c1c7 cells treated with SFN (positive control), MitoPQ, and MitoPQ Ctrl for 48 h (n = 8). The mean values are shown. *F*, induction of Nrf2 downstream targets (GCLC, GSS, HO-1) after treatment with 5-μM MitoPQ and MitoPQ Ctrl for 8, 12, 24 h. Protein levels were assessed by Western blotting for GCLC, GSS, HO-1, and GAPDH. Relative fold of induction was obtained as compared with the untreated/MitoPQ Ctrl and GAPDH. All data are the mean ± SD. Blots are representative of three independent experiments. *p* values were calculated using one-way ANOVA (Tukey’s post hoc correction for multiple comparisons) or two-tailed, unpaired Student’s T-test. Individual significant *p* values are shown. GCLC, glutamate–cysteine ligase catalytic subunit; GSS, GSH synthetase; HO-1, heme oxygenase-1; NQO1, NAD(P)H:quinone oxidoreductase-1; Nrf2, nuclear factor erythroid 2–related factor 2.

It has been previously reported that increasing mitochondrial oxidative damage and/or stress activates the Nrf2 pathway (28). It was further suggested that mitochondrial oxidative damage and/or stress activated Nrf2 through kinase-dependent mechanisms such as the macrophage-stimulating 1 and macrophage-stimulating 2 systems (49). However, it was unclear whether mitochondrial superoxide production alone could activate Nrf2 by generating hydrogen peroxide as a redox signal that was released from the organelle to the cytosol, or whether the putative redox signal was secondary to intramitochondrial alterations. Therefore, here we used the targeted redox cycler MitoPQ to generate superoxide selectively within mitochondria (40), without directly affecting other mitochondrial processes, or the cytosolic redox environment of C2C12 cells (45). The effects of MitoPQ were compared with its control compound, which is taken up by mitochondria within cells but does not generate superoxide (44). C2C12 cells were treated with 5-μM MitoPQ, a concentration that has been shown to robustly increase superoxide production within mitochondria, but not in the cytosol, in these cells (43), as was confirmed here by showing that MitoPQ did not induce oxidative stress within the cytosol (Fig. S3*A*). MitoPQ did not cause an increase in Nrf2 protein levels (Fig. 4*A*) nor was there any localization of Nrf2 to the nucleus assessed by cell subfractionation followed by immunoblotting (Fig. 4*B*), in contrast to the positive control SFN (Fig. S1), or by immunofluorescence microscopy (Fig. 4, *C*–*D*), compared with the positive control H_2_O_2_. Under these conditions, MitoPQ did not elicit any changes in NQO1 activity (Fig. 4*E*) or in the expression of the Nrf2 targets, GCLC, GSS and HO-1 (Fig. 4*F* and Fig. S3*B*). Increasing MitoPQ concentrations 5- to 10-fold did not enhance cell levels of Nrf2 (Fig. S3*C*), indicating that the lack of effect on Nrf2 was not due to insufficient MitoPQ. We conclude that under these conditions, the selective production of superoxide and hydrogen peroxide within the mitochondrial matrix does not activate Nrf2.

### Both MitoCDNB and CDNB lead to Nrf2 activation, with NAC reversing MitoCDNB-mediated Nrf2 activation

**Figure 5:**
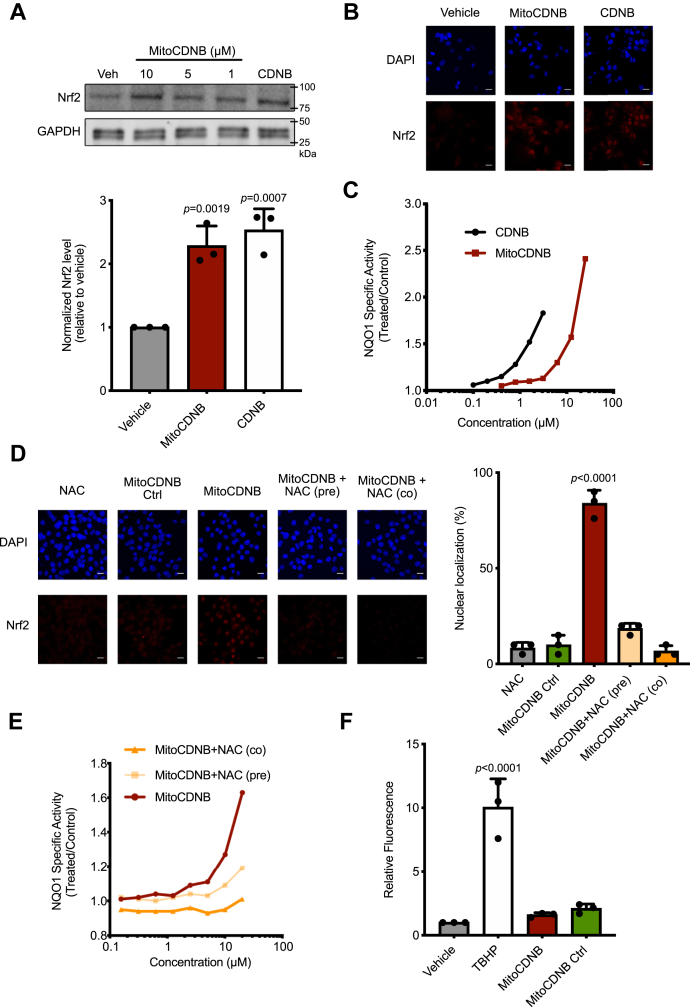
**MitoCDNB and CDNB comparison.***N*-Acetyl-cysteine diminishes MitoChlorodinitrobenzoic acid (MitoCDNB) effect on the Nrf2 pathway. *A*, Western blotting of Nrf2 protein levels. C2C12 cells were incubated with Ctrl (0.1% ethanol), MitoCDNB (10, 5, and 1 μM for 4 h), and CDNB (5 μM for 4 h). Protein levels were assessed by Western blotting for Nrf2 (top) and GAPDH (bottom). *B*, 3D maximum projection images showing fluorescence obtained with C2C12 cells with DAPI nuclear staining (*first row*) and immunocytochemistry for Nrf2 (*second row*). Nrf2 exhibits a cytosolic distribution, when C2C12 cells are incubated with a vehicle (0.1% ethanol) and a nuclear distribution when incubated with 10-μM MitoCDNB and 5-μM CDNB for 4 h. Scale bar: 20 μm. *C*, NQO1 activity in Hepac1c7 cells treated with MitoCDNB or CDNB for 24 h (n = 8). *D*, 3D maximum projection images showing fluorescence obtained with C2C12 cells with DAPI nuclear staining (*first row*) and immunocytochemistry for Nrf2 (*second row*). C2C12 cells are incubated with NAC (1 mM) and MitoCDNB Ctrl (10 μM) 4 h, whereas it exhibits a nuclear distribution with MitoCDNB (10 μM). After pretreatment or cotreatment with 1-mM NAC, a predominantly cytosolic distribution was observed. Scale bar: 20 μm. Quantification of nuclear distribution of Nrf2 in C2C12 cells in conditions described above. Nuclear distribution is presented as the mean percentage of all cells ± SD from 3 independent experiments, 30 cells were counted for each condition. *E*, NQO1 activity in mouse Hepa1c1c7 cells treated with MitoCDNB (10 μM), NAC (1 mM pretreatment) + MitoCDNB, or NAC (1 mM) + MitoCDNB (cotreatment) for 24 h (n = 8). *F*, cellular oxidative stress levels were assessed with CellROX using flow cytometry in C2C12 cells incubated with a vehicle (0.1% ethanol), *tert*-butyl hydroperoxide (250 μM—positive control), 10-μM MitoCDNB, or 10-μM MitoCDNB Ctrl for 4 h. *p* values were calculated using one-way ANOVA (Tukey’s post hoc correction for multiple comparisons) or two-tailed, unpaired Student’s T-test. All data are the mean ± SD. Blots are representative of three independent experiments. Individual significant *p* values are shown. NAC, *N*-acetyl-L-cysteine; NQO1, NAD(P)H:quinone oxidoreductase-1; Nrf2, nuclear factor erythroid 2–related factor 2.

From the previous results, we observed that MitoCDNB, but not MitoPQ, led to Nrf2 stabilization, nuclear localization, and enhanced expression of its downstream targets. Previously (42) it has been shown that MitoCDNB disrupts mitochondrial thiol antioxidant defenses and depletes mitochondrial GSH, which can disrupt mitochondrial thiol redox homeostasis, whereas the cytosolic GSH levels remained unchanged. In contrast, CDNB primarily disrupts cytosolic thiol antioxidant defenses (42). CDNB itself (5 μM) does increase Nrf2 protein levels (Fig. 5*A*) and its translocation to the nucleus (Fig. 5*B*) in C2C12 cells. Hepa1c1c7 cells exposed to CDNB and MitoCDNB both increased NQO1 activity (Fig. 5*C*). As MitoCDNB acts in part by depleting mitochondrial GSH levels, we next assessed the effect of NAC, a GSH precursor that also increases cell thiol levels and can thereby directly ameliorate cellular oxidative stress that impacts thiols (50), on Nrf2 activation by MitoCDNB. NAC (1 mM) added 1 h before, or simultaneously with, MitoCDNB (10 μM, 4 h) prevented the induction by MitoCDNB of Nrf2 nuclear localization (Fig. 5*D*). As expected, NAC did not have an influence on SFN-induced Nrf2 nuclear localization (Fig. S4*A*). The MitoCDNB-mediated induction of NQO1 was also diminished when cells were either pretreated (24 h) or cotreated with NAC (Fig. 5*E*). Treatment with NAC at concentrations up to 10 mM had no effect on the activity of NQO1 (Fig. S4*B*). As NAC and MitoCDNB do not interact directly (Fig. S5*A*), this suggests that NAC prevents the MitoCDNB-mediated Nrf2 activation by boosting thiol defenses within the cell. This was confirmed by showing that NAC did prevent depletion of whole-cell GSH by CDNB and MitoCDNB (Fig. S5*B*) and increased mitochondrial GSH even in the presence of MitoCDNB (Fig. S5*C*). To investigate further how MitoCDNB affected the cytosol, we used CellROX, which is a fluorescent probe that responds to a wide range of oxidative processes, enabling us to measure changes in whole-cell oxidative stress, but did not observe an increase with MitoCDNB (Fig. 5*F*).

### MitoCDNB activates Nrf2 *via* altering sensor thiols on Keap1

**Figure 6:**
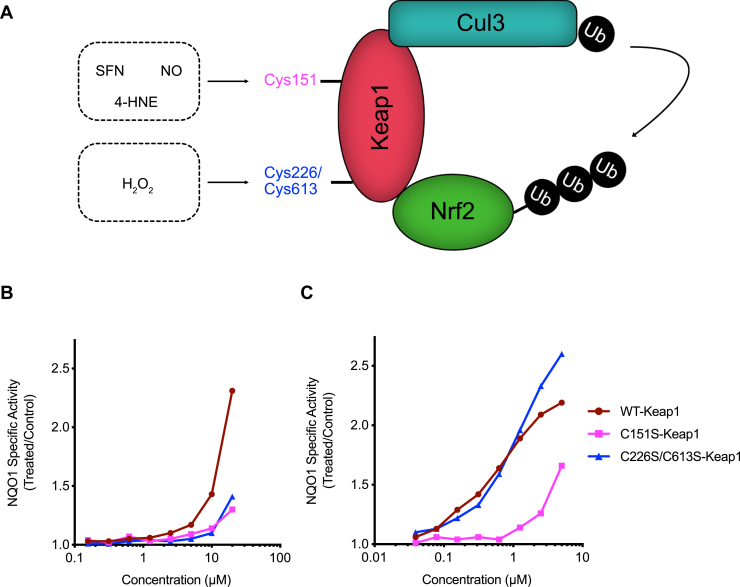
**The inducer activity of MitoCDNB is diminished in MEF cells expressing Keap1 cysteine mutants.***A*, a schematic representation of different classes of Nrf2 inducers acting on specific cysteines on Keap1, adapted from (41). *B*, NQO1 activity in Keap1^WT^, Keap1^C151S^, and Keap1^C226S/C613S^ MEF cells treated with MitoChlorodinitrobenzoic acid (MitoCDNB) for 24 h (n = 8). *C*, NQO1 activity in Keap1^WT^, Keap1^C151S^, and Keap1^C226S/C613S^ MEF cells treated with SFN for 24 h (n = 8). The mean values are shown. Keap1, Kelch-like ECH-associated protein 1; MEF, mouse embryonic fibroblast; NQO1, NAD(P)H:quinone oxidoreductase-1; Nrf2, nuclear factor erythroid 2–related factor 2; SFN, sulforaphane.

The above analysis indicates that MitoCDNB, but not MitoPQ, generates signals that activate Nrf2. Under homeostatic conditions, Nrf2 is bound to Keap1 and targeted for ubiquitination and proteolysis by the proteasome. The canonical pathway for the activation of Nrf2 by oxidants or electrophiles is *via* the reaction of the activators with thiols on Keap1, with specific thiols likely having particular reactivity with different species. These reactions disrupt the substrate adaptor function of Keap1 and enable Nrf2 to escape ubiquitination, migrate to the nucleus, and activate gene expression. To interrogate the involvement of Keap1 in the mechanism of Nrf2 activation by MitoCDNB, we used mouse embryonic fibroblast (MEF) cells expressing two different Keap1 cysteine mutants or their WT counterpart (41). Specifically, these mutants were Keap1^C151S^ and Keap1^C226S/C613S^ (Fig. 6*A*). Cys151 on Keap1 is the main sensor for SFN, 4-hydroxynonenal, and nitric oxide, whereas Cys226 and Cys613 respond to H_2_O_2_ (Fig. 6*A*) (51). The MEF cells were incubated with MitoCDNB, and NQO1 activity was measured 24 h later (Fig. 6*B*). In WT cells, MitoCDNB induced NQO1 in a concentration-dependent manner, but this induction was diminished in MEF cells expressing either of the Keap1 mutants (Fig. 6*B*). The NQO1 inducer potency of the classical Nrf2 activator SFN was also greatly reduced in the Keap1^C151S^ mutant cells in comparison with their WT or Keap1^C226S/C613S^ counterparts, in agreement with previous reports that Cys151 is the main sensor for SFN (7,52) (Fig. 6*C*). The use of Keap1 mutants confirms that MitoCDNB activates Nrf2 through multiple cysteine sensors in Keap1 and further suggests the involvement of both electrophiles and oxidants as potential mediators.

## Conclusions

Nrf2 plays a central role in the cytoprotective response to oxidative stress and is critical for the maintenance of mitochondrial redox homeostasis (13, 28). However, how mitochondrial oxidative stress and damage generate the signals that lead to Nrf2 activation in the cytosol is not understood. To address this, we used selective chemical biology approaches that enabled us to interrogate separately the effects of mitochondrial superoxide and hydrogen peroxide production and disruption of thiol redox homeostasis. We demonstrate that disrupting mitochondrial thiol redox homeostasis leads to Nrf2 activation, whereas enhanced mitochondrial superoxide production alone does not.

MitoCDNB has been previously shown to selectively deplete the mitochondrial GSH pool and inhibit TrxR2 in cells and *in vivo* (42). Our current results show that in C2C12 cells, MitoCDNB increases the levels of Nrf2, which then translocates to the nucleus (Fig. 2), inducing the expression of Nrf2 downstream targets such as HO-1, GCLC, GSS, and NQO1 (Fig. 3). By contrast, MitoPQ, a mitochondria-targeted redox cycler, does not activate Nrf2 (Fig. 4). MitoPQ generates superoxide selectively within mitochondria, which is in turn rapidly dismutated to H_2_O_2_ (43). The latter is formed locally and does not diffuse out of mitochondria within cells, which provides an explanation for why we do not observe Nrf2 activation. Furthermore, although there have been studies suggesting a link between mitochondrial hydrogen peroxide production and Nrf2 activation (28), such link has not been demonstrated directly. Our experiments with MitoPQ indicate that elevating superoxide and hydrogen peroxide within mitochondria, in the absence of other changes, is not capable of generating a hydrogen peroxide signal from the mitochondria to the cytosol to activate Nrf2. However, it is important to note that in disrupting the thiol-based antioxidant defenses within mitochondria, and peroxidases within mitochondria, MitoCDNB may lead indirectly to an elevation of mitochondrial hydrogen peroxide levels in the presence of other sources of superoxide (42). Thus, it may be that elevated mitochondrial hydrogen peroxide production, in conjunction with disruption of matrix thiol redox homeostasis, could lead to a mitochondria-generated hydrogen peroxide signal reaching the cytosol.

Our studies found that although CDNB is also an Nrf2 activator, as it increases the protein levels of Nrf2 and leads to its translocation to the nucleus (Fig. 5), it is much more toxic than its mitochondria-targeted counterpart MitoCDNB. This difference most likely arises because CDNB in the cytosol can affect global GSH levels both within the cytosol and also those within mitochondria because of the import of GSH from the cytosol to the mitochondria. Indeed, several studies have shown that global GSH depletion leads to activation of Nrf2 (53). It is also possible that CDNB may directly react with cysteines in Keap1; CDNB is a GST substrate, and GST substrates are known Nrf2 activators (54). In addition, NAC is generally regarded as a GSH precursor and therefore leads to an increase in GSH levels (50), but also increases levels of H_2_S (55), which may also contribute to its effects on MitoCDNB interactions with Nrf2 (56, 57). In the present study, we showed that pretreatment or cotreatment with NAC blocks the MitoCDNB-mediated stabilization and nuclear translocation of Nrf2, and transcription of its downstream targets (Fig. 5). Furthermore, as shown in Figure 3, Nrf2 activation through MitoCDNB increases transcription of enzymes involved in GSH metabolism: GCLC and GSS. Hence, our study suggests that mitochondrial GSH depletion is a signal for Nrf2 activation. This notion is also strengthened by the fact that MitoCDNB affects Nrf2 after 4 h, but not 1 h (Fig. 2), when the mitochondrial GSH depletion is well underway (42).

Here, we show that MitoCDNB activates Nrf2 through a Keap1-dependent mechanism that involves both Cys151 and Cys226/Cys613 sensors (Fig. 6). These residues of Keap1 have been shown to mediate Nrf2 activation by inducers such as SFN, 4-hydroxynonenal and H_2_O_2_ (41). At nontoxic concentrations, CDNB does not lead to any NQO1 induction in MEF cells expressing either WT or Keap1 cysteine mutants (data not shown), which suggests the effect of MitoCDNB is unrelated to any of its (minimal) cytosolic activity (Figs. 5 and 6). Our previous studies have shown that MitoCDNB leads to mitochondrial fragmentation (42), which suggests involvement of Drp1, and it is of interest that Nrf2 activation facilitates the proteasomal degradation of Drp1 (58). In addition, PGAM5 has been shown to act as a signaling hub, which brings the Keap1–Nrf2 complex to the mitochondria by forming a tripartite Keap1–PGAM5–Nrf2 complex (38, 39). Although MitoCDNB, has been shown to increase mitochondrial ROS (42), we did not see any increase in cellular oxidative stress (Fig. 5) or leakage of H_2_O_2_ from isolated mitochondria previously (42). However, this does not exclude the possibility of mitochondrial hydrogen peroxide acting on Keap1 in the close vicinity to the mitochondria.

In summary, we have shown that the selective disruption of mitochondrial thiol metabolism generates signals that then move from the mitochondria to the cytosol to interact with Keap1 cysteines to activate the Nrf2 pathway. The nature of these signals and how they exit mitochondria to interact with thiols on Keap1 will be explored in future studies.

## Experimental procedures

### Cell culture

C2C12 (mouse myoblast; European Collection of Authenticated Cell Cultures and MEF (41) cells were maintained at subconfluency (<80%) at 37 °C in a humidified atmosphere of 95% air and 5% CO_2_ in Dulbecco’s modified Eagle’s medium (DMEM; Gibco) with high glucose (4.5 g/l D-glucose), supplemented with 10% (v/v) fetal bovine serum, 100 U/ml penicillin, and 100 μg/ml streptomycin. Hepa1c1c7 cells (murine hepatoma cells; European Collection of Authenticated Cell Cultures) were grown at 37 °C in a humidified atmosphere of 95% air and 5% CO_2_ in α-minimum essential medium supplemented with 10% (v/v) heat- and charcoal-inactivated fetal bovine serum with no antibiotics.

### Cellular fractionation

Cells were grown up to 20 × 10^6^ in 150-mm dishes (Thermo Fisher Scientific). After appropriate incubations, cells were detached using trypsin, pelleted (150*g*, 3 min, RT), and resuspended in a 1-ml fractionation buffer (FB) (220-mM D-mannitol, 70-mM sucrose, 1-mM EDTA, 10-mM Hepes, pH 7.4, 4 °C). Cells were homogenized by being passed through a 27-gauge needle 10 times and a sample was taken as a whole-cell fraction. The homogenate was centrifuged (720*g*, 5 min, 4 °C), with the whole-cell supernatant stored, and the nuclear pellet was washed and passed through a 25-gauge needle 10 times. The subsequent nuclear homogenate was centrifuged again (720*g*, 5 min, 4 °C), with the supernatant discarded, and the pellet containing nuclei was kept as the nuclear fraction. The aforementioned whole-cell supernatant containing the mitochondria, membranes, and cytoplasm was recentrifuged (800*g*, 10 min, 4 °C), and the pellet was discarded, with this step repeated 3 times at which point no pellet was observed. The resulting supernatant was subsequently centrifuged (9000*g*, 10 min, 4 °C), collecting the supernatant (S) and resuspending the mitochondrial pellet (P) in 1 ml of the FB. The centrifugation step was repeated on the supernatant. The resuspended pellet (P) was centrifuged (9000*g*, 15 min, 4 °C), the supernatant was discarded, and the pellet was resuspended in 50-μl FB (crude mitochondrial fraction). The supernatant (S) underwent an ultracentrifugation step (109,000*g*, 1 h, 4 °C), and the resulting supernatant was collected as the cytosolic fraction. The protein concentration of each fraction was measured with a bicinchoninic acid (BCA) assay (Thermo Fisher Scientific).

### Microscopy

C2C12 cells were plated at 15,000 cells/well in 24-well plates containing glass coverslips. After 16 h, the compounds of interest were added. At the end of the incubation, the media was removed, cells were washed twice in cold PBS (Gibco), treated with 4% (w/v) paraformaldehyde for 15 min at 37 °C followed by three 5-min washes with PBS. Cells were then permeabilized with 0.1% (v/v) Triton X-100 in PBS for 10 min at RT, washed with PBS (3 × 10 min), blocked with 10% (w/v) bovine serum albumin (BSA) in PBS for 20 min at RT, and then incubated with primary antibody in 5% (w/v) BSA in PBS for 2 h at RT. The primary antibody solution was removed, and cells washed with PBS (3 × 10 min) and incubated with secondary antibodies in 5% (w/v) BSA in PBS for 1 h at RT. This solution was then removed, and the cells were washed with PBS (3 × 10 min). Coverslips were then mounted onto slides using Dako Mounting Medium (5 μl/coverslip; Agilent) and analyzed using Zeiss LSM880 confocal system equipped with a Zeiss Plan-Achromat 63x/1.4 NA oil-immersion objective. Z-stacks were acquired at 0.5-μm steps.

Representative images for each condition were taken from >30 cells. This process was repeated on three separate cell passages to ensure biological replication. Primary antibody was rabbit anti-Nrf2 (1:1000; Cell Signaling Technologies, number: 12721). The secondary antibody was AlexaFluor488 goat anti-rabbit (1:1000; Thermo Fisher Scientific, number: A-11008).

### Flow cytometry

C2C12 cells were seeded at 0.5 × 10^6^ cells per ml and treated as described previously. Thirty minutes before the end of the stimulation, CellROX (1 μM) was added directly into the cell culture medium. Cells were shielded from light with tinfoil and incubated at 37 °C for 30 min. Fifteen minutes before the end of CellROX incubation, SYTOX Red (1 μM) was added into the cell culture medium. Cells were washed with PBS, scraped in PBS (0.5 ml), and transferred to polypropylene fluorescence activated cell sorting tubes. At least 10,000 total events were acquired using a BD LSRFortessa cell analyzer (BD Biosciences). CellROX Green and SYTOX Red were excited by laser at 488 nm and 640 nm, and the data were collected at forward scatter, side scatter, 530/30 nm (CellROX), and 660/20 (SYTOX) detector. The data were analyzed using FlowJo software (version 10.4.2), with the cell debris, represented by forward and side scatters, gated out for analysis. The median fluorescence intensity of the 530/30 nm channel was measured as a measure of cellular oxidative stress.

### Western blotting

C2C12 cells were plated at 300,000 cells/well in 6 well plates (Thermo Fisher Scientific) and treated with test reagents after a day of growth. At the end of the incubation, the media was removed, and cells were washed twice in cold PBS (Gibco), before the addition of 100 μl of RIPA buffer (Merck) supplemented with protease inhibitors (cOmplete protease inhibitor cocktail, Merck) and 15-min incubation on ice. The samples were centrifuged at 17,000*g* for 10 min at 4 °C, and the supernatants were collected for protein quantification using the BCA assay (Thermo Fisher Scientific) with BSA as a standard. Samples were mixed with 4× loading buffer (200-mM Tris-Cl, pH (6.8), 8% (w/v) SDS, 0.4% (w/v) bromophenol blue, 40% (v/v) glycerol), 400-mM DTT as a reductant. Typically, 10 to 40 μg of protein was loaded onto 12% or 4 to 20% Tris-Glycine SDS-PAGE gels (Bio-Rad) and run at 120 V for 1 h. Protein was transferred to polyvinylidene difluoride membranes (Immobilon-FL or Bio-Rad Trans-Blot Turbo Mini) by wet transfer (in the presence of 25-mM Tris, 192-mM glycine, 20% (v/v) methanol, pH (8.4)) or semidry transfer (Bio-Rad Trans-Blot Turbo) before blocking for 1 h at RT with Odyssey blocking buffer (LI-COR). Primary antibody incubation was completed in 4% (v/v) Odyssey buffer in PBS + 0.1% (v/v) Tween-20 (PBST) overnight at 4 °C. Membranes were then washed 3 × 15 min in PBST, followed by secondary antibody incubation in 4% Odyssey buffer in PBS for 1 h at RT, washed 2 × 15 min in PBST and 1 × 15 min in PBS. Membranes were then visualized using a LI-COR Odyssey CLx system or enhanced chemiluminescence (Amersham ECL Prime Western blotting reagent) and analyzed using Image Studio Lite. Primary antibodies: rabbit anti-GAPDH (1:5000, Sigma, number: G9545), rabbit anti-Nrf2 (1:1000, Cell Signaling Technologies, number: 12721), mouse anti–histone-4 (1:1000, Cell Signaling Technologies, number: 2935), mouse anti-Tubulin (1:1000, Abcam, number: ab56676), rabbit anti-GCLC (1:1000, Abcam, number: ab190685), rabbit anti-GSS (1:1000, Abcam, number: ab133592), and mouse anti–HO-1 (1:1000, Abcam, number: ab13248). Secondary antibodies were goat anti-rabbit IRDye800 (1:10,000, LI-COR, number: 926–32211) and goat anti-mouse IRDye680 (1:10,000, LI-COR, number: 926–68070), or goat anti-rabbit IgG HRP conjugate (1:3000, Promega, number: W4011) and goat anti-mouse IgG HRP conjugate (1:3000, Promega, number: W4021).

### GSH measurement

To determine the total GSH pool (GSH + 2 × GSSG) whole cells (1 × 10^6–9^ cells) or mitochondrial fractions (isolated with the hypotonic method as described (42)) were treated with 100 μl 5% (w/v) sulfosalicylic acid with vortexing for 30 s, proteins precipitated by centrifugation (16,000*g* for 10 min), and the supernatant analyzed by the GSH recycling assay as described (59, 60). Data are expressed as GSH equivalents (GSH + 2 × GSSG) normalized to protein determined by the BCA assay.

### NQO1 assay

Inducer potency was quantified by use of the NQO1 bioassay in Hepa1c1c7 mouse hepatoma cells (46, 47). Cells (1 × 10^4^ per well of a 96-well plate) were grown for 24 h and exposed (n = 8) to serial dilutions of compounds for the time periods indicated in the figure legends. NQO1 enzyme activity was quantified in cell lysates using menadione as a substrate. Protein concentrations were determined in aliquots from the same cell lysates by the BCA assay (Thermo Scientific). The CD value was used as a measure of inducer potency. The same assay was used to determine the NQO1 enzyme activity in MEF cells, but the starting number of cells was 2 × 10^4^ per well.

### Mitochondrial isolation

For isolated mitochondria studies, mitochondria were prepared from rat livers. In all cases, mitochondria were isolated by homogenization using a Dounce homogenizer followed by differential centrifugation at 4 °C. Liver mitochondria were prepared in STE buffer (250-mM sucrose, 10-mM Tris, 1-mM EGTA, pH 7.4). Homogenates were pelleted by centrifugation at 3000*g* for 3 min. The supernatant was collected and centrifuged at 10,000*g* for 10 min. Mitochondrial pellets were resuspended in 10 ml and recentrifuged at 10,000*g* for 10 min before final resuspension in 5 ml per liver. In all cases, the protein concentration was measured using the BCA assay with BSA as a standard.

### Mitochondrial incubations

Mitochondrial incubations were in KCl buffer (120-mM KCl, 10-mM Hepes, 1-mM EGTA, pH 7.2) at 37 °C, with succinate (10 mM), rotenone (4 μg/ml), and carbonyl cyanide 4-(trifluoromethoxy)phenylhydrazone (0.5 μM) added as appropriate, unless stated otherwise. After incubations, mitochondria were pelleted by centrifugation (7500*g* for 10 min) and pellets and supernatants analyzed as necessary. Supernatants were immediately snap-frozen, whereas pellets were extracted in 250-μl acetonitrile (ACN) + 0.1% TFA before centrifugation (16,000*g*, 10 min, 4 °C), and the resulting supernatant snap-frozen.

### Reversed-phase HPLC

Samples for reversed-phase HPLC were prepared by diluting to 25% (v/v) ACN containing 0.1% TFA, followed by centrifugation (16,000*g* for 10 min) and filtration of the supernatant through a syringe-driven 0.22-μm polyvinylidene difluoride filter unit (Millex, Millipore). Samples were then loaded into a 2-ml sample loop and separated by reversed-phase HPLC using a C18 column (Jupiter 300A, Phenomenex) attached to a Wide Pore C18 guard column (Phenomenex), all driven by a Gilson 321 pump. A flow rate of 1 ml/min was used with a gradient of 0.1% (v/v) TFA in water (buffer A) and 0.1% (v/v) TFA in ACN (buffer B) at (%B), 0 to 2 min; 5%, 2 to 17 min; 5 to 100%, 17 to 19 min; 100%, 19 to 22 min; 100 to 5%. Absorbance was measured at 220 (triphenylphosphonium) and 340 (GSH adduct) nm using a UV-Visible detector (Gilson 151).

### Synthesis of MitoCDNB control

Diisopropylamine (598 μl, 3.34 mmol, 2.0 eq) was added to a stirred solution of 5-chloro-2,4-dinitrotoluene (397 mg, 1.83 mmol, 1.1 eq) and (4-aminobutyl)triphenylphosphonium bromide (692 mg, 1.67 mmol, 1.0 eq) in dry ACN (10 ml) at RT under argon. The solution was stirred overnight and poured into 1 M hydrochloric acid and extracted into CH_2_Cl_2_ (30 ml). The organic layer was washed with 1 M hydrochloric acid (2 × 100 ml), dried over sodium sulfate, and concentrated under vacuum. The residue was purified by column chromatography using a 12 g Agela cartridge eluting with CH_2_Cl_2_:MeOH (100:0 increasing to 90:10 over 10 column volumes) to give the chloride salt of MitoCDNB control as a yellow solid foam (638 mg, 69%) (Fig. S6). δ_H_ (400 MHz: CDCl_3_): 1.74 to 1.84 (2H, m, PCH_2_C*H*_*2*_), 2.08 to 2.15 (2H, m, NCH_2_C*H*_*2*_), 2.71 (3H, s, Me), 3.80 (2H, m, NHC*H*_*2*_), 4.05 to 4.12 (2H, m, PCH_2_), 7.25 (1H, s, H-6), 7.64 to 7.69 (6H, m, ArH), 7.74 to 7.79 (3H, m, ArH), 7.86 to 7.91 (6H, m, ArH), 8.29 (1H, t, *J* = 5.1 Hz, NH), 8.97 (1H, s, H-3). δ_C_ (101 MHz: CDCl_3_): 19.83 (d, *J* = 3.9 Hz, CH_2_), 22.27 (d, *J* = 50.6 Hz, CH_2_), 22.27 (CH_3_), 29.07 (d, *J* = 17.1 Hz, CH_2_), 42.44 (CH_2_), 117.83 (CH), 118.34 (d, *J* = 85.9 Hz, C), 125.79 (CH), 128.59 (C), 130.60 (d, *J* = 12.6 Hz, CH), 133.84 (d, *J* = 10.0 Hz, CH), 135.15 (d, *J* = 3.0 Hz, CH), 137.06 (C), 143.81 (C), 147.25 (C). δ_P_ (162 MHz: CDCl_3_): 24.66 (s). m/z (ESI): Found: 514.1881. C_29_H_29_O_4_N_3_P requires *M*^*+*^, 514.1890.

### Statistical analysis

Statistical comparison between two groups was carried out using GraphPad Prism 7 software using two-tailed, unpaired Student’s t-tests or one-way ANOVA with the appropriate correction for multiple comparisons. The number of biological replicates (n) and statistical values are stated in each figure legend. n = number of replicates. Individual significant *p* values are shown in the figures (statistical significance corresponds to *p* < 0.05).

## Data availability

All data from this study are contained within the manuscript, including Supplemental Information.

## Conflict of interest

The authors declare that they have no conflicts of interest with the contents of this article.
