# Supplementary material for: Nrf2 is activated by disruption of mitochondrial thiol homeostasis but not by enhanced mitochondrial superoxide production
Source: J Biol Chem. 2020 Dec 13;296:100169. doi: 10.1074/jbc.RA120.016551 (PMC7948991; doi:10.1074/jbc.RA120.016551)
Supplement: Figures S1–S6 [file mmc1.pdf]

SUPPORTING INFORMATION FOR

Nrf2 is activated by disruption of mitochondrial thiol homeostasis but not by enhanced mitochondrial superoxide production

Filip Cvetko[a], Stuart T. Caldwell[b], Maureen Higgins[c], Takafumi Suzuki[d], Masayuki Yamamoto[d,e], Hiran A. Prag[a], Richard C. Hartley[b], Albena T. Dinkova-Kostova[c,f], Michael P. Murphy[a,g,1]

From the [a]MRC Mitochondrial Biology Unit, University of Cambridge, Keith Peters Building, Cambridge Biomedical Campus, Cambridge, CB2 0XY, UK; [b]School of Chemistry, University of Glasgow, Glasgow G12 8QQ, UK; [c]Jacqui Wood Cancer Centre, Division of Cellular Medicine, School of Medicine, University of Dundee, Dundee, Scotland DD1 9SY, UK; [d]Department of Medical Biochemistry, Tohoku University Graduate School of Medicine, 2-1 Seiryo-machi, Aoba-ku, Sendai 980-8575, Japan; [e]Tohoku Medical Megabank Organization, Tohoku University, 2-1 Seiryo-machi, Aoba-ku, Sendai 980-8573, Japan; [f]Department of Pharmacology and Molecular Sciences and Department of Medicine, Johns Hopkins University School of Medicine, Baltimore, MD21205, US; [g]Department of Medicine, University of Cambridge, Cambridge Biomedical Campus, Cambridge, CB2 0QQ, UK

Running title: Nrf2 activation by disruption of mitochondrial thiols

[1]To whom correspondence should be addressed: Professor Michael P. Murphy, MRC Mitochondrial Biology Unit, Hills Road, Cambridge CB2 0XY, UK, Phone: +44 1223 252 900 mpm@mrc-mbu.cam.ac.uk

Keywords: reactive oxygen species (ROS); MitoPQ; MitoCDNB; Nrf2; redox signaling; thiol oxidation; energy metabolism

---

List of Contents:

Supporting Figures S1-S6

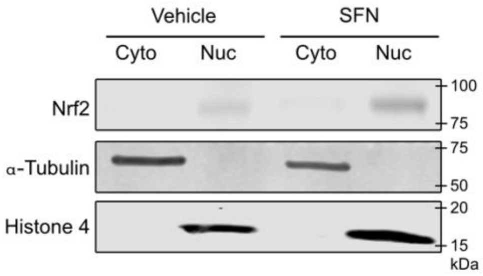

Figure S1. SFN leads to Nrf2 nuclear localisation.
C2C12 cells were incubated for 4 h with either Vehicle (0.1% Ethanol) or 5 µM SFN and fractionated into cytosolic and nuclear fractions. Protein levels were then assessed by western blotting for Nrf2 (top), alpha-Tubulin (middle) and Histone-4 (bottom). Blots are representative of three independent experiments.

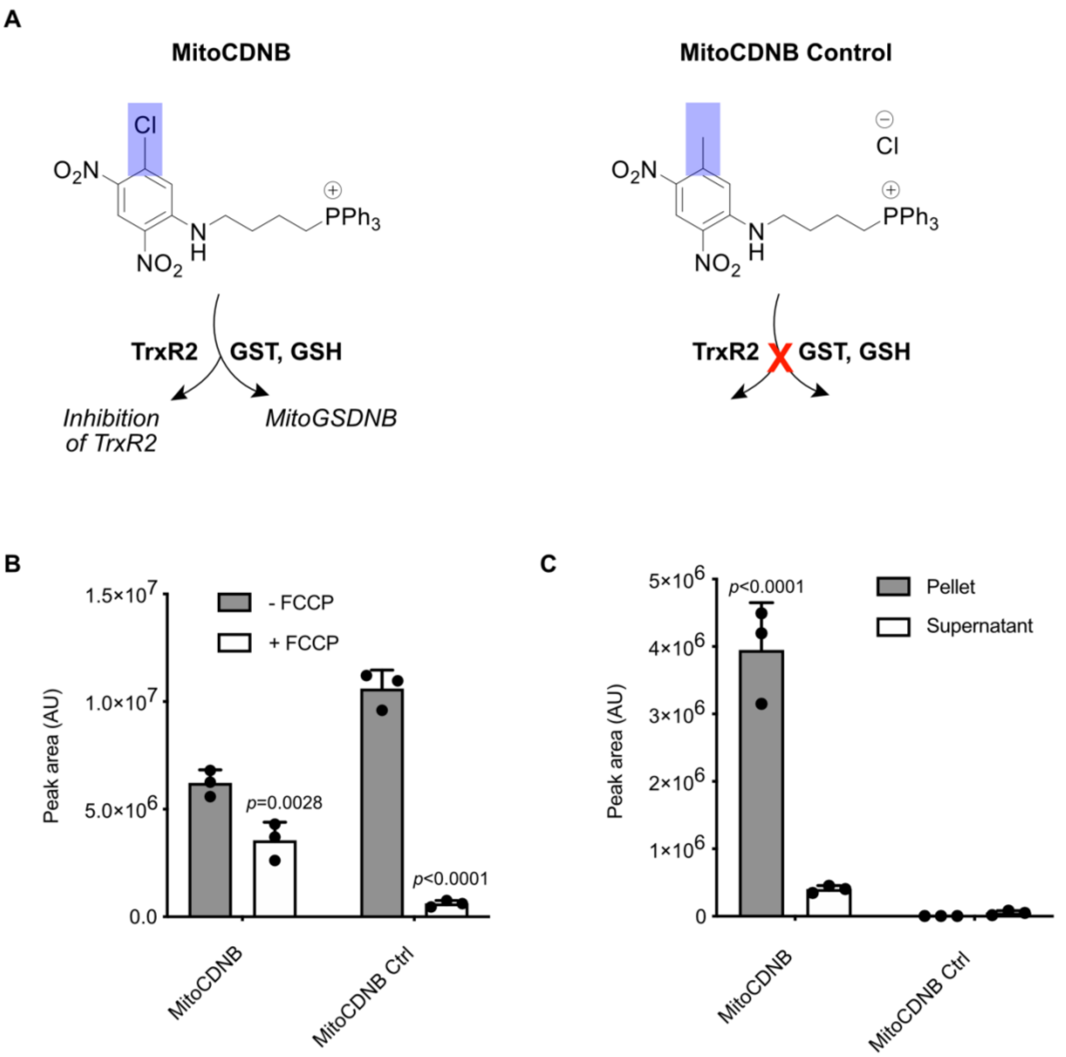

Figure S2. Comparison of MitoCDNB and MitoCDNB Control.

A, the Cl- leaving group on CDNB's moiety of MitoCDNB (blue bracket), is missing in MitoCDNB Control, replaced with a methyl group, which is non-reactive. This leads to no reaction with GST, GSH or TrxR2. *B*, uptake by isolated mitochondria. Rat liver mitochondria (1 mg/ml) were incubated in KCl buffer with succinate (10 mM), rotenone (4 µg/ml), TPMP (5 µM), and MitoCDNB or MitoCDNB Control (both at 5 µM) +/- FCCP (0.5 µM) at 37 °C for 5 min and the mitochondrial pellet analyzed for TPP-containing compounds by RP-HPLC with peak area recorded. *C*, the peak area of GSH adducts from samples in (*B*) were also measured by RP-HPLC and peak areas quantified. All data are mean ± SD. *P* values were calculated using one-way ANOVA (Tukey's post hoc correction for multiple comparisons) or two-tailed, unpaired Student's t-test. Individual significant p values are shown (statistical significance corresponds to $p < 0.05$).

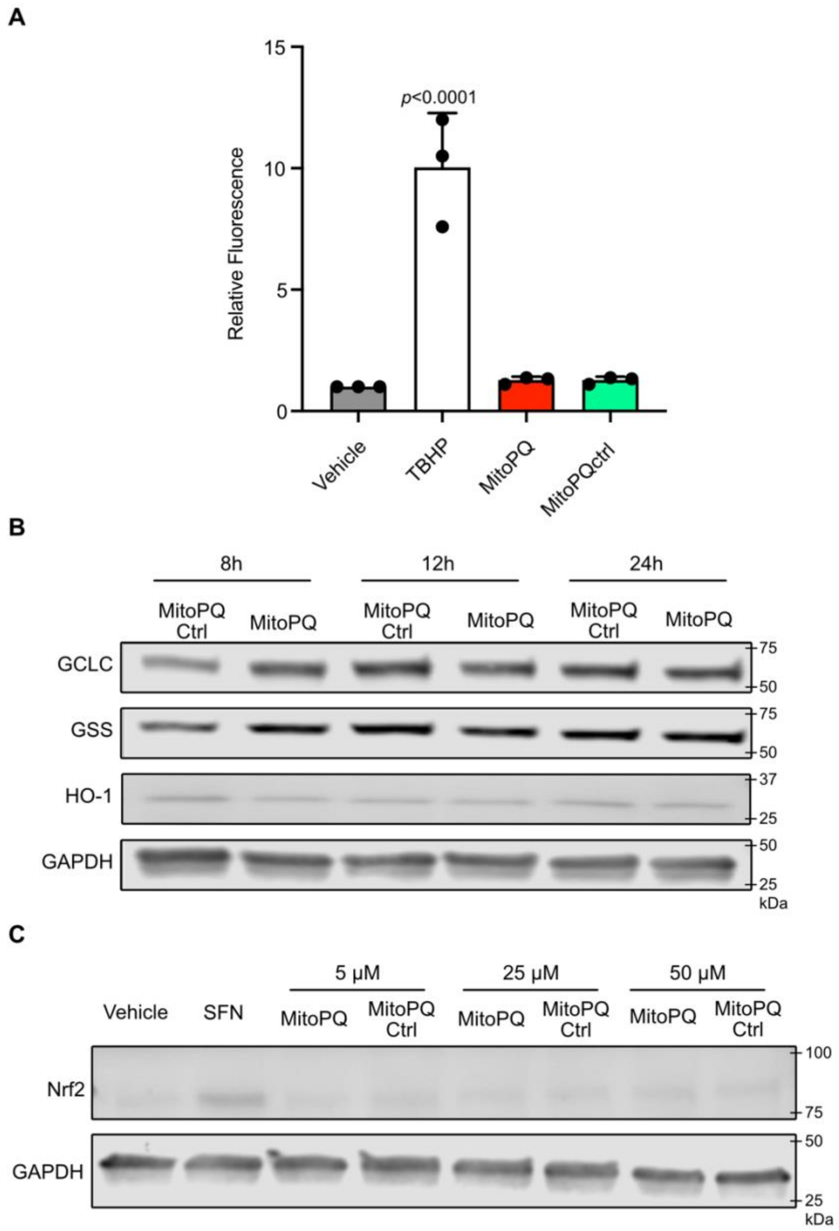

Figure S3. MitoPQ does not induce cytosolic oxidative stress or Nrf2 activation.
A, cellular H_2O_2 levels were assessed with CellROX using flow cytometry in C2C12 cells incubated with vehicle (0.1% ethanol), TBHP (250 µM - positive control), MitoPQ (5 µM) or MitoPQ Ctrl (5 µM) for 4 h. *B*, western blotting of Nrf2 downstream targets (GCLC, GSS, HO-1) after treatment with 5 µM MitoPQ and MitoPQ Ctrl for 8,12 or 24 h. Protein levels were assessed by western blotting for GCLC, GSS, HO-1 and GAPDH. *C*, western blotting of Nrf2 protein levels. C2C12 cells were incubated with Vehicle (0.1% ethanol), SFN (5 µM) and MitoPQ (5, 25, 50 µM for 4 h) and MitoPQ Ctrl (5, 25, 50 µM for 4 h). Protein levels were then assessed by western blotting for Nrf2 (top) and GAPDH (bottom). Blots are representative of three independent experiments. All data are mean ± SD. *P* values were calculated using one-way ANOVA (Tukey's post hoc correction for multiple comparisons) or two-tailed, unpaired Student's t-test student T-test. Individual significant p values are shown (statistical significance corresponds to $p < 0.05$).

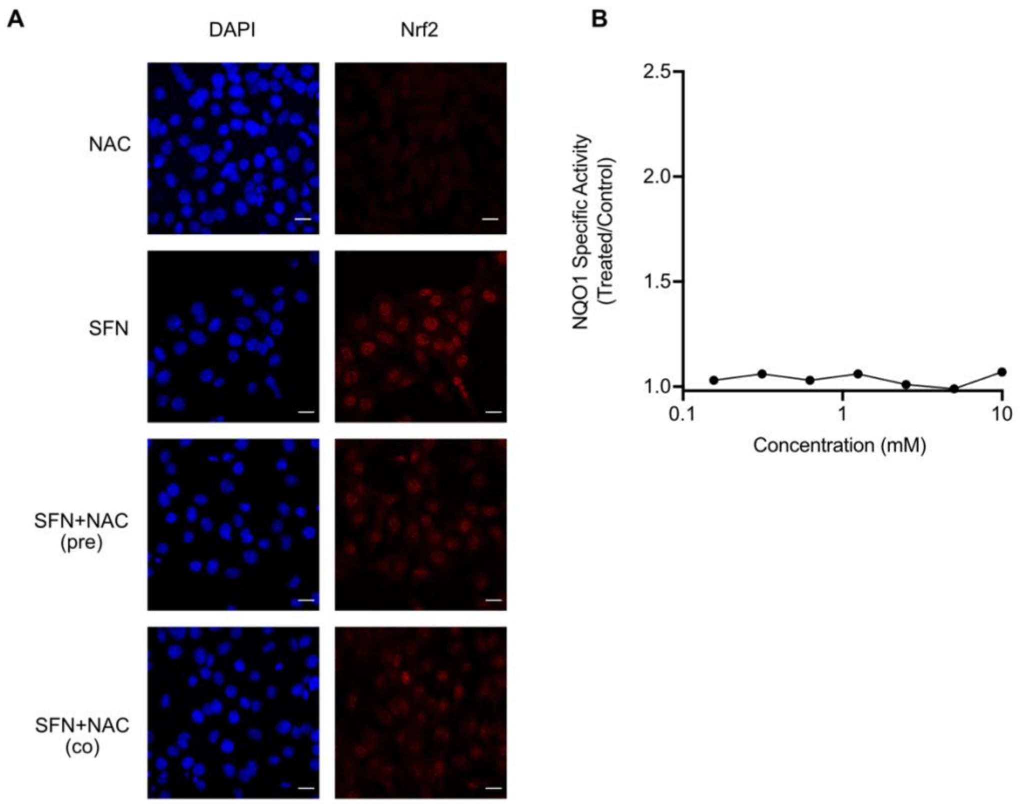

Figure S4. NAC does not prevent Nrf2 activation by SFN and has no effect on NQO1 activity. *A*, 3D maximum projection images showing fluorescence obtained with C2C12 cells with DAPI nuclear staining (first column), immunocytochemistry for Nrf2 (second column). C2C12 cells are incubated with NAC (1mM) and SFN (5 µM) for 4 h. A nuclear distribution is seen with SFN (5 µM). After pre-treatment or co-treatment with NAC (1mM) the nuclear distribution stays the same. Scale bar: 20 µm. *B*, NQO1 activity in mouse Hepa1c1c7 cells treated with NAC for 24 h (n = 8). Mean values are shown.

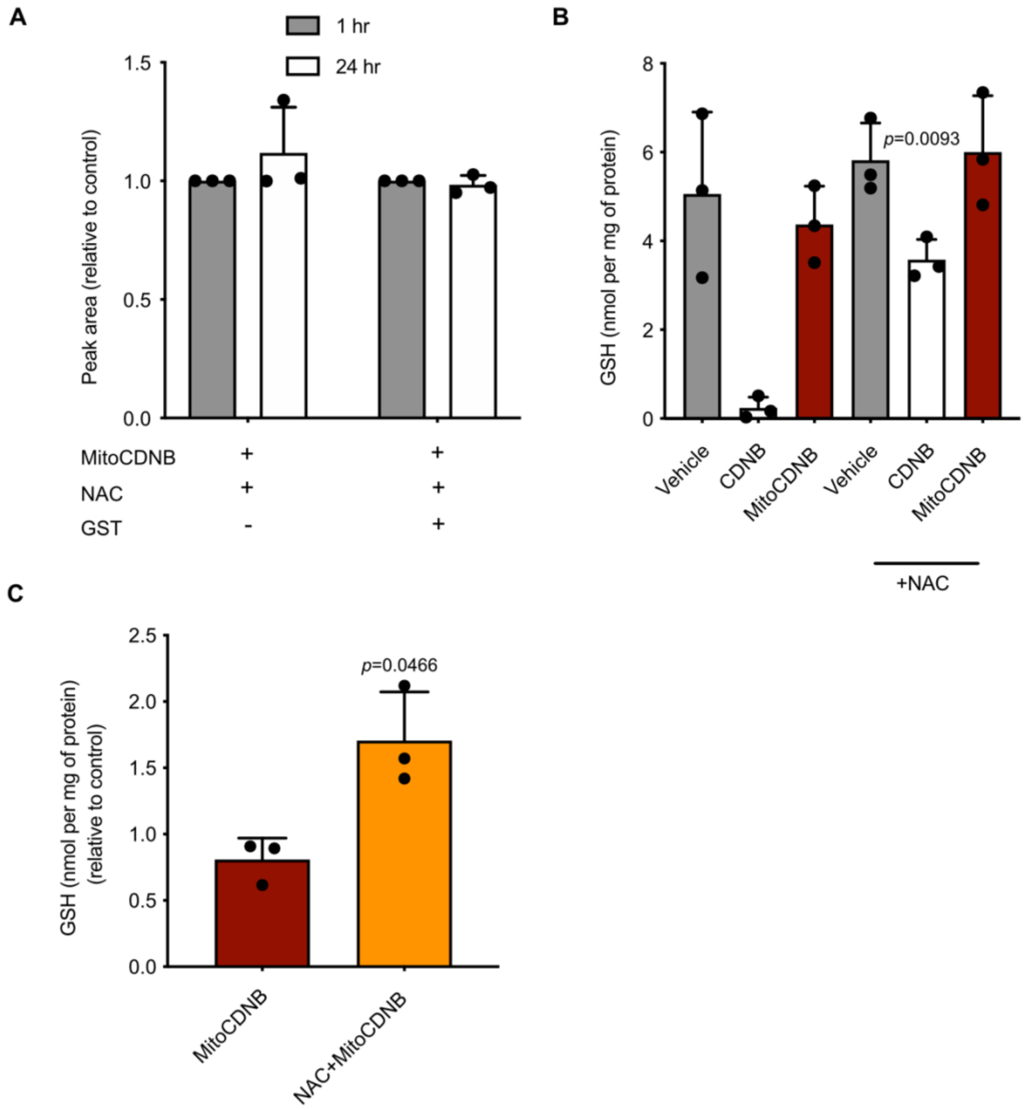

Figure S5. N-acetyl cysteine increases cellular and mitochondrial GSH.
A, NAC does not react with MitoCDNB. MitoCDNB (10 µM) and NAC (10 mM) were incubated in KCl buffer with and without GST (10 µg) for 1 and 24 h before being analyzed by RP-HPLC with MitoCDNB peak area recorded. The peak areas were normalized to the peak area of the internal standard TPMP (10 µM). *B*, rescue of cell GSH with NAC. C2C12 cells were incubated for 1 hr with Vehicle (0.1% ethanol), CDNB (10 µM) and MitoCDNB (10 µM) +/- NAC (1 mM). *C*, rescue of mitochondrial GSH with NAC. C2C12 cells were incubated with MitoCDNB (10 µM) +/- NAC (1 mM), mitochondria were then isolated and GSH measured. All data are mean ± SD. *P* values were calculated using one-way ANOVA (Tukey's post hoc correction for multiple comparisons) or two-tailed, unpaired Student's t-test student T-test. Individual significant p values are shown (statistical significance corresponds to $p < 0.05$).

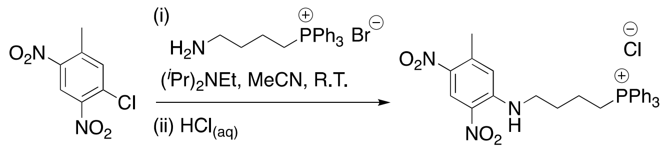

MitoCDNB Control 69%

Figure S6. Synthesis of MitoCDNB Control.
MitoCDNB Control was synthesized using 5-chloro-2,4-dinitrotoluene and (4-aminobutyl)triphenylphosphonium bromide in the presence of diisopropylamine, dry acetonitrile at RT under argon and stirred overnight. After extraction and subsequent concentration, the residue was purified by column chromatography to give the phosphonium salt as a yellow solid foam (69%).
